# Liraglutide exhibits anti-inflammatory activity through the activation of the PKA/CREB pathway

**DOI:** 10.1186/s12950-019-0225-z

**Published:** 2019-10-11

**Authors:** Li Wang, Linjie Feng, Jingyu Zhang

**Affiliations:** 1 Department of Hand Surgery, The Second Hospital of Tangshan, No. 21 Jianshe Road, Tangshan, Hebei, 063000 Tangshan, Hebei People’s Republic of China; 2 Department of Orthopedics, North China University of Science and Technology Affiliated Hospital, Tangshan, Hebei People’s Republic of China

**Keywords:** GLP-1R, Liraglutide, PKA/CREB pathway, Inflammation, Osteoarthritis

## Abstract

Qihong Que and colleagues found that liraglutide exhibited anti-inflammatory activity through the activation of the PKA/CREB pathway in an OA rat model. We believe there was a flaw in this research. In their first experiment, the sacrifice time of the 10 rats in the control group has not been stated. And when the rats in the OA-1, OA-5, OA-10, OA-20 and OA-28 groups were sacrificed, they were in different weeks of age. If the rats in the control group were compared to the rats in the OA-1, OA-5, OA-10, OA-20 and OA-28 groups respectively, the results may be biased due to differences in the week age of the rats. We believe that addressing this issue could further increase the value of their study.

## Main text

Qihong Que. and colleagues [1] found that liraglutide exhibited anti-inflammatory activity through the activation of the PKA/CREB pathway in an OA rat model. We believe there was a flaw in this research. In their first experiment, the sacrifice time of the 10 rats in the control group has not been stated. And when the rats in the OA-1, OA-5, OA-10, OA-20 and OA-28 groups were sacrificed, they were in different weeks of age. If the rats in the control group were compared to the rats in the OA-1, OA-5, OA-10, OA-20 and OA-28 groups respectively, the results may be biased due to differences in the week age of the rats. We believe that addressing this issue could further increase the value of their study.

We declare no competing interests.

## Conclusions

Qihong Que. and colleagues found that liraglutide exhibited anti-inflammatory activity through the activation of the PKA/CREB pathway in an OA rat model. We believe there was a flaw in this research.

## Data Availability

Not applicable.

## Abbreviations

CREB: cyclic adenosine monophosphate (cAMP) response element-binding
OA: osteoarthritis
PKA: protein kinase A
